# Investigating the Relationship between Oral Health and Severe Mental Illness: Analysis of NHANES 1999–2016

**DOI:** 10.3390/dj12070191

**Published:** 2024-06-24

**Authors:** Jing Kang, Jianhua Wu, Vishal. R. Aggarwal, David Shiers, Tim Doran, Jasper Palmier-Claus

**Affiliations:** 1 Faculty of Dentistry, Oral, and Craniofacial Sciences, King’s College London, London SE1 9RT, UK; 2 Centre for Primary Care, Wolfson Institute of Population Health, Queen Mary University of London, London E1 4NS, UK; 3 School of Dentistry, University of Leeds, Leeds LS2 9JT, UK; 4 Division of Psychology & Mental Health, University of Manchester, Manchester M13 9PL, UK; david.shiers@doctors.org.uk; 5 Health Services & Policy, University of York, York YO10 5DD, UK; tim.doran@york.ac.uk; 6 Spectrum Centre for Mental Health Research, Division of Health Research, Lancaster University, Lancaster LA1 4YW, UK; j.palmier-claus@lancaster.ac.uk; 7 Lancashire & South Cumbria NHS Foundation Trust, Lancashire PR5 6AW, UK

**Keywords:** severe mental illness, National Health and Nutrition Examination Survey (NHANES), oral health, tooth loss, tooth decay, periodontal disease, psychosis

## Abstract

Objectives: To explore whether: (i) people with severe mental illness (SMI) experience worse oral health than the general population, and (ii) the risk factors for poor oral health in people with SMI. Methods: Cross-sectional data were extracted from the National Health and Nutrition Examination Survey (1999–2016), including on self-rated oral health, oral pain, tooth loss, periodontitis stage, and number of decayed, missing, and filled teeth. Candidate risk factors for poor oral health included demographic characteristics, lifestyle factors, physical health comorbidities, and dental hygiene behaviours. Ordinal logistic regression and zero-inflated negative binomial models were used to explore predictors of oral health outcomes. Results: There were 53,348 cases included in the analysis, including 718 people with SMI. In the fully adjusted model, people with SMI were more likely to suffer from tooth loss (OR 1.60, 95% CI: 1.34–1.92). In people with SMI, risk factors identified for poor oral health outcomes were older age, white ethnicity, lower income, smoking history, and diabetes. Engaging in physical activity and daily use of dental floss were associated with better oral health outcomes. Conclusions: People with SMI experience higher rates of tooth loss than the general population, and certain subgroups are particularly at risk. Performing regular physical exercise and flossing may lower the risk of poor oral health, while smoking and diabetes may increase the risk. These findings suggest opportunities for targeted prevention and early intervention strategies to mitigate adverse oral health outcomes in people with SMI.

## 1. Introduction

Poor physical health in people with severe mental illness (SMI; e.g., schizophrenia, bipolar disorder) is a major research priority [1]. Public Health England defined SMI as “people with psychological problems that are often so debilitating that their ability to engage in functional and occupational activities is severely impaired” [2], typically including people experiencing psychotic disorders or bipolar disorder. One important, but often neglected, area of investigation is the disparity in oral health [3,4,5]. People with SMI may experience worse oral health than the general population [6], with higher rates of decayed, missing, and filled teeth (DMFT) [7]. In some cases, poor oral health can interfere with basic functions like eating [8], thus profoundly affecting quality of life [9] and limiting employment opportunities [10]. There is a major need to understand oral health inequalities in people with SMI to develop effective and targeted interventions. 

The reasons for poor oral health in people with SMI are complex. There is some indication that people with SMI are less likely to brush their teeth or own a toothbrush than the general population [11], and unhealthy lifestyles like smoking [12], drug use [13], and poor diet [14]. Xerostomia is a common side effect of psychotropic medication and can elevate the risk of tooth decay, infections by human immunodeficiency virus or hepatitis C, and salivary gland aplasia, leading to diminishing quality of life [15,16]. Access to treatment may also be limited in people with SMI, with one study in Denmark suggesting that only a third of patients will attend an annual dental appointment [17,18]. There is evidence that both cardiovascular disease (CVD) and diabetes are associated with poor oral health [19]. Not only are these health conditions highly prevalent in people with SMI [20], but, when combined with poor oral health, they can add to the burden of multiple morbidity.

To date, research on oral health outcomes in SMI has mostly employed small samples and, with some exceptions, has failed to control for relevant clinical, lifestyle, and demographic covariates [7]. They have also tended to focus on levels of decayed, missing or filled teeth (DMFT), rather than a broader range of oral health outcomes (e.g., mouthache, periodontal disease) and self-reported oral health [21]. One study using the UK Biobank data showed worse self-reported periodontal disease in people with SMI than in the general population, but there was a lack of clinical validation of oral data and the participants were restricted to an older population [4]. Furthermore, little is known about the factors contributing to poor oral health in people with SMI. Wey and colleagues [22] found that the DMFT score was associated with older age and duration of untreated illness in inpatients with psychosis, but oral health self-care behaviours, lifestyle factors, and other candidate risk factors for poor oral health were not included in their analysis. 

In this study, clinically assessed oral health outcomes in people with SMI using a large, nationally representative sample from the National Health and Nutrition Examination Survey (NHANES) [23] were examined. The aim was to explore whether people with SMI have worse oral health than people without SMI. Risk factors for poor oral health in people with SMI were also assessed. 

## 2. Methods

Study design: The study followed STROBE guidelines. Study participants came from the cross-sectional NHANES 1999–2016. A detailed description of NHANES can be found in the supplement. 

Study participants: Data from nine NHANES surveys (1999–2016) were extracted. The resulting sample from NHANES for participants over 18 years old was 53,348 participants (25,709 men and 27,639 women). NHANES does not contain clinical diagnoses of mental illness, so participants’ prescription medicines in the past month were extracted and people with SMI were identified based on the type of medication that they were taking. If participants reported taking one or more medications for treating SMI, they were considered to have SMI. More details on medication can be found in Supplement Methods.

Oral health outcome measures: Oral health outcomes included dentition (number of teeth; 0, edentulous; 32, full dentition), dental caries (number of decayed, missing and filled teeth, 0–32), periodontal status (none, mild, moderate and severe), and self-reported oral health status (excellent, very good, good, fair, and poor). More details on oral health outcomes can be found in the supplement.

Exposures and covariates: The main exposure was SMI status among the general population, and the covariates include demographics, anthropometric, lifestyle, comorbidities (the simultaneous presence of two or more diseases or medical conditions in a patient), and dental hygiene behaviors.

When investigating predictors of poor oral health among people with SMI alone, the exposures were demographics, anthropometric, lifestyle, comorbidities, and dental hygiene behaviors. More details on those can be found in the supplement, and directed acyclic graphs were presented in Figure S1 to illustrate the relationship between exposures and oral health outcomes.

Statistical analyses: Descriptive statistics were used for people with and without SMI, concerning demographics, anthropometrics, lifestyles, comorbidities, dental hygiene behaviour, and all oral health outcomes (dentition, dental caries, periodontal status, and self-reported oral health status). Continuous variables were presented as means (SD) or medians (interquartile range), and categorical variables were reported as frequencies (%). The general population was matched to people with SMI on a 3 to 1 ratio based on age and gender because the distribution for people with and without SMI was different in the original dataset (people with SMI were of older age than people without SMI) and matching the sample could provide comparable results. For example, a male participant with SMI of age 61 years old was randomly matched with three male participants without SMI of the same age. Matching cases with controls is commonly used in epidemiolocal studies, because it can increase statistical efficiency without introducing bias. The results presented using matched techniques are easier to explain and are more intuitive for clinicians. 

For statistical modelling, self-rated oral health was further grouped as ‘excellent or very good or good’ and ‘fair or poor’, ache in the mouth as ‘never or hardly ever’ and ‘occasionally to very often’, and periodontal status was further grouped as ‘none’, ’mild to moderate’, and ’severe’ to ensure sufficient events in each category. Smoking was grouped into two categories (non-smoker, ever smoker) as were dental visits (fewer than 1 per year, more than 1 per year or never). Alcohol and energy intake were excluded in the statistical model because they were not significantly different between people with and without SMI. Education was highly correlated with family income and, therefore, only the latter was included in the models. Carbohydrate intake was also highly correlated with sugar intake and, therefore, only sugar intake was included in the model. Cigarette numbers only applied to smokers, so it was excluded from the modelling process. Tooth-brushing frequency had only a small number of responses (<1% of the total sample) in both groups and was excluded. The ordinal variables of dentition (tooth loss) and periodontal status necessitated ordinal regression models. Self-reported oral health (self-rated oral health status, mouthache) were binary so logistic regression models were applied. First, a univariable model with one of the ordinal oral health outcomes as the dependent variable and group (SMI or non-SMI) as the independent variable was performed. Secondly, multivariable models were performed with gradual adjustment of demographic, lifestyles, comorbidities, and dental hygiene behaviours. Similarly, zero-inflated negative binomial (ZINB; see supplement) models were used to compare SMI and non-SMI population on dental caries (DMFT, DT and MT), because dental caries variables have excess zeros and follow a negative binomial distribution. A ZINB model is a 2-part model, with the logit model predicting excessive zeros and negative binomial model predicting the counts [24]. Similar approaches for the univariable ZINB model and multivariable ZINB model were applied for DMFT, DT, and MT, respectively. 

When investigating predictors of oral health status in people with SMI alone, ordinal regression, logistic regression, and ZINB models were used for ordinal, binary, and scale oral outcomes, respectively, with similar approaches. Missing data were imputed five times through multiple imputations by chained equations according to the distribution of the imputed variables. Pooled modelling estimates and accompanying standard errors (SE) were generated according to Rubin’s rules [25]. Data was also analyzed on complete case and sensitivity analyses were conducted. Statistical analyses were performed in R version 3.4.1 (https://cran.r-project.org/, accessed on 7 June 2023. R is developed by Posit Software, Boston, MA, US) with various packages (e.g., MASS, MICE, Amelia, accessed on 7 June 2023).

## 3. Results

**Figure 1:**
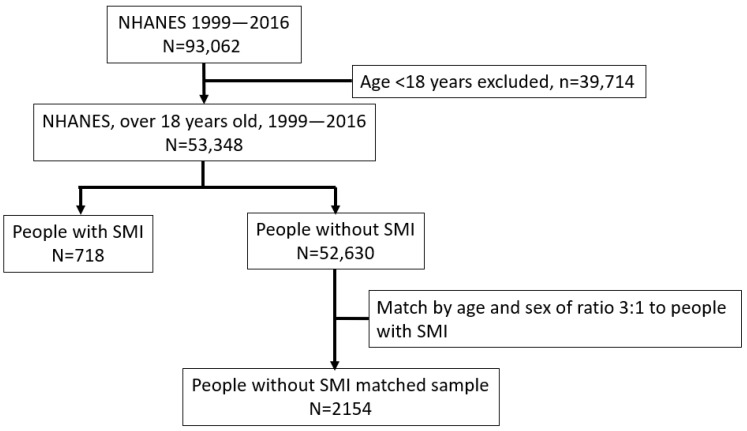
Flowchart of the participants’ selection.

There were 53,348 participants included in the analyses (Figure 1). The average age of the total sample was 47.5 years (SD 19.6 years) and 25,709 (48.2%) were men. 

### 3.1. Characteristics between People with and without SMI 

Table 1 shows the demographic and lifestyle information of the participants. The mean age of people with SMI was 51.1 (SD 16.8) years, higher than the general population (47.4 years, SD 19.6). A higher proportion of people with SMI were white (52.2% vs. 43.7%), and a lower proportion had a higher education degree (41.3% vs. 48.6%). More people with SMI were unmarried (70.6% vs. 49.3%) and their family income was lower on average (ratio of income to poverty 1.8 vs. 2.5). On average, people with SMI had a higher BMI (30.5 vs. 28.6), greater waist circumference (68.2% vs. 52.9%) and were substantially more likely to be current smokers (38.3% vs. 19.4%) than people without SMI. More people with SMI suffered from substance misuse (35.7% vs. 17.4%), were less physically active (30.1% vs. 43.2%), consumed more sugar (137.6 vs. 114.7 g) and carbohydrates (273.8 vs. 254.9 g), and were more likely to suffer from diabetes (18.2% vs. 11.0%) and cardiovascular disease (16.7% vs. 9.0%).

### 3.2. Oral Health in People with and without SMI 

For oral hygiene behaviours, there were similar rates of dental visiting between people with and without SMI, but people with SMI used dental floss daily less frequently (18.6% vs. 31.7%). Due to the low response of tooth brushing frequency in people with SMI (*n* = 3), no conclusions could be drawn for this variable (Table 2).

For oral health outcomes, people with SMI were more likely to rate their oral health as ‘poor’ (23.1% vs. 12.2%), experience mouthache (8.8% vs. 3.4%), and be edentulous (14.5% vs. 6.7%). They also experienced higher levels of tooth decay (DMFT median 1.5 vs. 1.0), but no differences in levels of periodontal disease (Table 2).

Table 3 shows the association between SMI status and poor oral health outcomes using the matched case-control samples of people with and without SMI (ratio 1:3 matched by age and sex). With full adjustment of demographic lifestyles, comorbidities and oral hygiene behaviour, people with SMI were more likely to experience tooth loss (OR = 1.60, 95% CI 1.34–1.92), mouthache (OR = 1.42, 95% CI 1.11–1.82), and a higher number of dental caries (RR = 1.14, 95% CI 1.05–1.23). Periodontal disease severity, self-rated oral health, and the chance to be caries-free or have full dentition were not associated with SMI status. 

Sensitivity analysis on complete cases showed similar results. 

### 3.3. Risk Factors of Poor Oral Health in People with SMI

Lastly, risk factors for poor oral health outcomes in the SMI sample were explored. Older age (OR 1.08, 95% CI: 1.06–1.09), smoking history (OR 2.70, 95% CI 1.76–4.13), and diabetes (OR 1.86, 95% CI: 1.07–3.26) were associated with higher levels of tooth loss. Conversely, higher family income (OR 0.76, 95% CI: 0.65–0.88), being physically active (OR 0.60, 95% CI: 0.39–0.94), and using dental floss everyday (OR 0.51, 95% CI: 0.28–0.91) were associated with lower levels of tooth loss. Higher family income was found to be associated with less risk of mouthache (OR 0.74, 95% CI: 0.59–0.92) and poor self-rated oral health (OR 0.84, 95% CI:0.72–0.98). Engaging in physical activity halved the risk of poor self-rated oral health (OR 0.51, 95% CI 0.31–0.82). In terms of dental caries, older age, white ethnicity, family income, being a smoker, and not using dental floss everyday were associated with a higher DMFT score. Full dentition (no missing teeth, zero-inflated part of the model) was associated with younger age (OR 0.96, 95%CI: 0.94–0.98), white ethnicity (OR 1.76, 95% CI 1.09–2.77), and higher family income (OR 1.31, 95% CI 1.08–1.58). Older age, white ethnicity, smoker history, and diabetes were associated with a higher number of missing teeth (negative binomial part of the model, RR (95% CI): 1.04 (1.03–1.06), 1.57 (1.15–2.16), 1.79 (1.24–2.58), 1.52 (1.03–2.24), respectively) while using dental floss everyday was associated with fewer missing teeth (RR 0.52, 95% CI: 0.34–0.79). No associations were identified for periodontal disease severity potentially due to the smaller sample size for this outcome. The effect sizes and significance levels remained stable when various modelling strategies were applied with different covariates to test the robustness of the results. Table 4 shows the risk factors mentioned above, and Table S1 shows the full list of risk factors tested.

## 4. Discussion

This study investigated the oral health of people with SMI using a large national dataset. The results showed that people with SMI suffered with more teeth lost due to dental disease and that modifiable risk factors for their poorer oral health included smoking and poor oral hygiene. 

People with SMI were 40% more likely to have lost teeth than the general population and 15% of the sample were edentulous. Tooth loss is an end stage of periodontal disease, dental cavity, and other dental disease when preventive or conservative treatments fail [26]. Tooth loss can cause difficulties with essential functions such as eating and speaking [27]. Additionally, poor oral health can lead to disfigurement, acute and chronic infection, eating and sleeping disruption, hospitalization and lost work [28]. 

No evidence of disparity in periodontal disease severity between people with and without SMI in this study was found. This might be due to the limited sample size in the NHANES 1999–2004, 2009–2014 cohorts that included periodontal measurements—periodontal status was only available in 283 (39%) participants with SMI. In another study using UK Biobank data^4^, it was found that SMI and periodontal disease were strongly associated. 

This study identified subgroups of people with SMI who might be at higher risk of poor oral health, including those of a white ethnicity and lower family income. This may be evidence of health intersectionality whereby certain characteristics combine to particularly disadvantage subsections of the population, which has clear relevance for oral health [29,30,31,32]. Lifestyle behaviours were also related to poor oral health. For example, smoking increased the risk of tooth loss by 2.6 times and dental caries by more than 20%. There is strong evidence to suggest that people with SMI are more likely to smoke and smoke more heavily than the general population [12,33], and the findings provide evidence of its harmful effect on oral health, consistent with other studies [34,35]. This study showed that doing moderate physical exercise every week and using dental floss every day was associated with better oral health. This association has also been reported elsewhere [36,37]. It is possible that exercise may lead to greater satisfaction with general and mental health, which extends to oral health. Furthermore, physical exercise may protect against inflammatory processes that act as mediators of physical disorders, which may also apply to oral disorders [38,39]. It is also possible that people who live healthy and physically active lifestyles generally are also more likely to attend to their teeth and gums and less likely to smoke, drink alcohol, and eat sugary foods. The benefit of flossing may offer additional protection to teeth and gums, over brushing alone [40,41], but might also have acted as a proxy for established oral hygiene behaviours more generally. 

The association between tooth loss and diabetes was unsurprising given that diabetes may contribute to dental problems, especially if blood glucose control is poor, adversely affecting oral bacterial flora and leading to tooth decay and gum disease [19,42,43]. This may, in part, reflect a shared exposure to common risk factors that include smoking, poor nutrition, obesity and physical inactivity. However, causality is likely to be complex; there may be a two-way relationship in which poor glycaemic control increases the risk of periodontitis, and periodontal inflammation adversely affects glycaemic control [44]. Older age was found to be associated with tooth loss and dental caries. Therefore, the early phase of mental illness may present a critical time to prevent progression to the poor oral health observed in older people. 

This study used a proxy measure of SMI in prescriptions of antipsychotic or mood-stabilizing medication, which is a limitation for generalizing our findings. Ascertainment of diagnosis in large cohort studies is complex with variable levels of overlap between cases identified through prescribed medications and other approaches, such as data linkage, self-report, and symptom-based measures [45]. The overlap between medication and formal diagnostic constructs in the current dataset is unclear. The study method lacks the advantage of triangulation between diagnostic categorization, record linkage, and medication. Using medication as an indicator of SMI might underestimate or overestimate the prevalence of SMI. For example, this NHANES dataset may have identified patients with first-episode psychosis as SMI who do not meet the full ICD diagnostic criteria for schizophrenia. Conversely, some patients with a diagnosis of schizophrenia may decline treatment with antipsychotic medication. In a previous study [4], it was estimated that only 48% of patients who were clinically diagnosed with SMI were taking psychosis-related medication, while 32% of people who were taking medication were clinically diagnosed, suggesting moderate levels of overlap.

Also, due to small numbers and missing data in some of the years of NHANES data collection, it was not possible to investigate the impact of polypharmacy, which may cause side effects that adversely affect oral health. Psychological (e.g., dental anxiety) and systemic (e.g., access to services) risk factors also require investigation in relation to poor oral health in SMI. Another limitation of this study is that it is unknown whether people with SMI were independent and autonomous in their daily routines or taken care of by caregivers. 

This study highlighted the importance of tackling oral health inequalities in people with SMI. In the United Kingdom, rehabilitation guidelines for complex psychosis [46] recommend that psychiatric services signpost support oral health, but levels of adherence are unclear. The results suggested that oral health inequalities extend to community samples, which also needs recognition in broader mental health and dental policies and guidelines. To date, interventions around oral health in SMI have largely focused on education. The Three Shires Trial [47] evaluated brief dental awareness training for early intervention in psychosis staff, which had no significant impact on any outcome. Other small feasibility studies have suggested that education interventions may improve oral health outcomes in SMI when combined with behavioral change techniques [48]. Little is known about the effectiveness of such interventions when rolled out into clinical practice and research is needed in this area. 

This study suggested that risk profiling for poor oral health is possible in people with SMI. For example, people with diabetes and smokers may be at particular risk and may benefit from additional support and signposting around their oral health. There exist focused evidence-based smoking cessation programmes for people with SMI [33], which require greater uptake across healthcare providers. Lastly, the effect of age on oral health outcomes indicates opportunities for early detection and intervention for younger people in the early phases of mental illness. 

## 5. Conclusions

People with SMI are more likely to experience tooth loss than people without SMI. Risk factors for poor oral health in SMI included older age, white ethnicity, lower income, smoking history, and diabetes. Physical activity and daily use of dental floss were associated with better oral health outcomes. Overall, the findings highlight oral health inequalities in people with SMI.

## Figures and Tables

**Table 1:** Characteristics of participants with and without SMI NHANES (*n* = 53,348), 1999–2016.

Characteristic	Total	Without SMI	With SMI	*p*-Value
*n*	53,348	52,630	718	
Age in years, mean (SD)	47.5 (19.6)	47.4 (19.6)	51.1 (16.8)	<0.001
Men	25,709 (48.2)	25,378 (48.2)	331 (46.1)	0.275
Ethnicity, white	23,388 (43.8)	23,013 (43.7)	375 (52.2)	<0.001
Education, college or above	23,974 (48.5)	23,691 (48.6)	283 (41.3)	<0.001
Marital status, not married	25,418 (49.6)	24,923 (49.3)	495 (70.6)	<0.001
Ratio of family income to poverty, mean (SD)	2.5 (1.6)	2.5 (1.6)	1.8 (1.4)	<0.001
BMI (kg/m^2^), mean (SD)	28.6 (6.8)	28.6 (6.7)	30.5 (7.6)	<0.001
Waist circumference ^a^				<0.001
Low	13,215 (27.5)	13,112 (27.7)	103 (17.3)	
High	9291 (19.4)	9204 (19.4)	87 (14.6)	
Very high	25,473 (53.1)	25,066 (52.9)	407 (68.2)	
Had at least 12 alcohol drink in one year	30,261 (69.4)	29,865 (69.4)	396 (67.9)	0.454
Smoking status				<0.001
Non-smoker	30,575 (57.3)	30,305 (57.6)	270 (37.6)	
Ex-smoker	12,278 (23.0)	12,105 (23.0)	173 (24.1)	
Current smoker	10,495 (19.7)	10,220 (19.4)	275 (38.3)	
Cig number in past 30 days, mean (SD)	12.6 (10.6)	12.5 (10.5)	16.4 (12.8)	<0.001
Substance Misuse (cocain/herion)	5766 (17.6)	5602 (17.4)	164 (35.7)	<0.001
Had moderate Physical activity over past 30 days	22,558 (43.0)	22,353 (43.2)	205 (30.1)	<0.001
Sugar intake (g), mean (SD)	115.0 (69.7)	114.7 (69.4)	137.6 (87.1)	<0.001
Carbohydrate intake (g), mean (SD)	255.1 (115.5)	254.9 (115.4)	273.8 (124.1)	<0.001
Energy intake (KCAL), mean (SD)	2060.7 (883.4)	2059.8 (883.2)	2132.0 (890.4)	0.060
Diabetes	5821 (11.1)	5695 (11.0)	126 (18.2)	<0.001
Cardiovascular disease ^b^	4869 (9.1)	4749 (9.0)	120 (16.7)	<0.001

Note: Data are presented as frequency (%) unless specified. Numbers may not sum to totals due to missing values; percentages may not sum to 100 due to rounding. SD, standard deviation; BMI, body mass index; KCAL, kilocalories. ^a^ Waist circumference is defined as low (men ≤ 94 cm, women ≤ 80 cm), high (men 94–102 cm, women 80–88 cm), very high (men > 102 cm, women > 88 cm). ^b^ cardiovascular disease is defined as having at least one of the congestive heart failure, coronary heart disease, angina, heart attack, and stroke *p*-value was obtained from comparing characteristics between people with and without SMI using appropriate statistical tests (*t* test/Mann–Whitney U test for continuous data and Chi-squared/Fisher’s exact test for categorical data).

**Table 2:** Oral hygiene behaviour and oral health status of participants with and without SMIs. NHANES (*n* = 53,348), 1999–2016.

Oral Characteristic	Total	Without SMI	With SMI	*p*-Value
*n*	53,348	52,630	718	
Oral hygiene behaviour				
Last dental visit				0.250
Less than a year	15,736 (54.1)	15,536 (54.1)	200 (54.2)	
Over 1 year	12,648 (43.5)	12,483 (43.5)	165 (44.7)	
Never	693 (2.4)	689 (2.4)	4 (1.1)	
Toothbrush, twice or more	457 (76.2)	454 (76.4)	3 (50.0)	0.303
Use dental floss in the past 7 days				<0.001
No	6866 (36.3)	6666 (35.9)	200 (58.0)	
Not everyday	6107 (32.3)	6026 (32.4)	81 (23.5)	
Everyday	5953 (31.5)	5889 (31.7)	64 (18.6)	
Oral health status				
Self-rated oral health				<0.001
Excellent or very good	13,255 (28.9)	13,121 (29.0)	134 (21.0)	
Good	16,348 (35.6)	16,160 (35.7)	188 (29.5)	
Fair	10,665 (23.2)	10,497 (23.2)	168 (26.4)	
Poor	5665 (12.3)	5518 (12.2)	147 (23.1)	
Ache in mouth				<0.001
Very often	900 (3.5)	863 (3.4)	37 (8.8)	
Fairly often	1118 (4.3)	1081 (4.2)	37 (8.8)	
Occasionally	3971 (15.3)	3901 (15.3)	70 (16.6)	
Hardly ever	5617 (21.7)	5535 (21.7)	82 (19.4)	
Never	14,291 (55.2)	14,095 (55.3)	196 (46.4)	
Tooth loss				<0.001
No loss	28,522 (53.5)	28,247 (53.7)	275 (38.3)	
1–10	12,854 (24.1)	12,661 (24.1)	193 (26.9)	
11–20	5319 (10.0)	5238 (10.0)	81 (11.3)	
21–31	3020 (5.7)	2955 (5.6)	65 (9.1)	
Edentulus	3633 (6.8)	3529 (6.7)	104 (14.5)	
DMFT, median [IQR]	1.0 [0.0, 11.0]	1.0 [0.0, 11.0]	1.5 [0.0, 16.0]	0.001
DT, median [IQR]	0.0 [0.0, 5.0]	0.0 [0.0, 5.0]	0.0 [0.0, 3.8]	0.005
MT, median [IQR]	0.0 [0.0, 2.0]	0.0 [0.0, 2.0]	0.0 [0.0, 5.0]	<0.001
Periodontal diseases				0.093
None	12,487 (53.5)	12,342 (53.5)	145 (50.9)	
Mild	1913 (8.2)	1891 (8.2)	22 (7.7)	
Moderate	4486 (19.2)	4415 (19.1)	71 (24.9)	
Severe	4476 (19.2)	4429 (19.2)	47 (16.5)	

Note: Data are presented as frequency (%) unless specified. Numbers may not sum to totals due to missing values; percentage may not sum to 100 due to rounding. IQR, interquartile range; DMFT, number of decayed, missing, and filled teeth; DT, number of decayed teeth; MT, number of missing teeth. *p* value was for comparing dental behaviours and outcomes between people with and without SMI using appropriate statistical tests (*t* test/Mann–Whitney U test for continuous data and Chi-squared/Fisher’s exact test for categorical data).

**Table 3:** Association between severe mental illness status and oral health outcomes, NHANES (matched *n* = 2872), 1999–2016.

	Ordinal Oral Health Outcomes (OR, 95% CI) ^a^				Numeric Oral Health Outcomes (95% CI)					
					DMFT		DT		MT	
Models	Self-Rated Oral Health ^d^	Ache in Mouth ^e^	Tooth Loss ^f^	Periodontal Disease Severity ^g^	Zero-Inflated Model, OR	Count Model, RR	Zero-Inflated Model, OR	Count Model, RR	Zero-Inflated Model, OR	Count Model, RR
Sample size *n*	2514	1533	2872	1302	2872		2872		2872	
Unadjusted	1.84 *** (1.58–2.16)	1.75 *** (1.43–2.15)	1.93 *** (1.69–2.21)	1.11 (0.89–1.39)	0.89 (0.70–1.13)	1.28 *** (1.18–1.38)	1.45 *** (1.17–1.78)	1.02 (0.93–1.12)	0.49 *** (0.33–0.71)	1.37 *** (1.17–1.60)
Adjusted for ^b,c^										
+demographics	1.61 *** (1.37–1.90)	1.53 *** (1.24–1.88)	1.69 *** (1.46–1.94)	1.02 (0.81–1.29)	0.97 (0.77–1.23)	1.22 *** (1.15–1.29)	1.27 * (1.03–1.56)	1.05 (0.96–1.14)	0.64 (0.38–1.05)	1.41 *** (1.23–1.62)
+lifestyles	1.19 (0.97–1.47)	1.46 ** (1.14–1.87)	1.66 *** (1.39–1.99)	0.77 (0.59–1.01)	1.08 (0.74–1.58)	1.16 *** (1.08–1.26)	1.60 *** (1.19–2.15)	1.02 (0.93–1.13)	0.99 (0.59–1.67)	1.39 *** (1.18–1.64)
+comorbidities	1.18 (0.96–1.45)	1.43 ** (1.12–1.83)	1.63 *** (1.36–1.95)	0.77 (0.58–1.01)	1.09 (0.74–1.60)	1.16 ** (1.07–1.25)	1.57 ** (1.17–2.11)	1.03 (0.93–1.13)	1.01 (0.60–1.71)	1.37 ** (1.16–1.61)
+oral hygiene behaviour	1.19 (0.97–1.47)	1.42 ** (1.11–1.82)	1.60 *** (1.34–1.92)	0.76 (0.58–1.01)	1.10 (0.75–1.61)	1.14 * (1.05–1.23)	1.54 (1.14–2.07)	1.02 (0.92–1.12)	0.99 (0.59–1.65)	1.30 ** (1.10–1.52)

^a^ OR estimates and 95% CI were pooled over the 5 imputed datasets. ^b^ logistic regression or ordinal logistic regression models were performed for ordinal or binary oral health outcomes to assess the inequality of oral health outcomes comparing people with and without SMI. Models are incrementally adjusted for demographics (age, sex, ethnicity, marital status, income), lifestyles (BMI, smoking status, substance misuse, physical activity, sugar intake), comorbidities (diabetes, cardiovascular disease), and oral health behaviour (dental visit, dental floss use). ^c^ Zero-inflated negative binomial (ZINB) models were performed to assess the inequality of dental caries experience comparing people with and without SMI. ZINB model is a 2-part model, with zero-inflated model predicting the chance of excessive zeros, and the count model predicts the number of DMFT/DT/MT. For example, in DMFT unadjusted model, zero-inflated odds ratio 0.94 indicated that people with SMI has 6% (1–0.94) lower chance of being caries-free (DMFT = 0), and the count model RR as 1.17 showed that people with SMI have 17% higher chance to have more dental caries than people without SMI. ^d^ self-rated oral health: excellent to good (ref), fair to poor. ^e^ Mouthache: never or hardly ever (ref), occasionally to very often. ^f^ tooth loss: no loss (ref), 1–10, 11–20, 21–31, and edentulous. ^g^ periodontal disease severity: none (ref), mild to moderate, severe. OR, odds ratio; RR, rate ratio; DMFT, number of decayed, missing and filled teeth; DT, number of decayed teeth; MT, number of missing teeth due to decay. * *p* < 0.05, ** *p* < 0.01, *** *p* < 0.001.

**Table 4:** Significant risk factors of poor oral health in people with severe mental illness, NHANES (*n* = 718), 1999–2016.

	Ordinal Oral Health Outcomes, OR (95% CI)				Numeric Oral Health Outcomes, OR and RR (95% CI)			
	Self-Rated Oral Health	Ache in Mouth	Tooth Loss Number (Grouped)	Periodontal Disease Severity	DMFT		MT	
					OR (95% CI)	RR (95% CI)	OR (95% CI)	RR (95% CI)
*n*	637	422	718	285	718		718	
Demographics								
Age	1.01 (0.99–1.03)	1.00 (0.97–1.03)	1.08 *** (1.06–1.09)	1.09 *** (1.05–1.13)	0.95 * (0.91–0.98)	1.03 *** (1.02–1.04)	0.96 *** (0.94–0.98)	1.04 *** (1.03–1.06)
Ethnicity, white	1.13 (0.74–1.73)	1.35 (0.79–2.30)	1.38 (0.96–1.98)	0.61 (0.32–1.17)	1.17 (0.49–2.78)	1.20 ** (1.03–1.40)	1.76 ** (1.09–2.77)	1.57 ** (1.15–2.16)
Ratio of family income to poverty	0.84 * (0.72–0.98)	0.74 ** (0.59–0.92)	0.76 *** (0.65–0.88)	0.70 * (0.53–0.94)	1.22 (0.88–1.68)	0.91 * (0.86–0.97)	1.31 * (1.08–1.58)	0.82 (0.72–0.93)
Lifestyles								
Smoking status, ever smoker	1.37 (0.85–2.21)	0.68 (0.38–1.22)	2.70 *** (1.76–4.13)	1.31 (0.61–2.82)	0.50 (0.20–1.25)	1.25 * (1.05–1.49)	0.72 (0.42–1.24)	1.79 ** (1.24–2.58)
Physical activity, yes	0.51 ** (0.31–0.82)	1.33 (0.74–2.38)	0.60 * (0.39–0.94)	0.79 (0.35–1.77)	1.02 (0.40–2.59)	0.99 (0.82–1.18)	0.85 (0.51–1.43)	0.93 (0.66–1.33)
Comorbidities								
Diabetes, yes	1.69 (0.90–3.17)	1.12 (0.55–2.28)	1.86 * (1.07–3.26)	1.42 (0.57–3.49)	1.85 (0.57–5.98)	1.15 (0.93–1.44)	0.79 (0.41–1.51)	1.52 * (1.03–2.24)
Dental hygiene behaviour								
Use dental floss in the past 7 days								
No (ref)	1	1	1	1	1	1	1	1
Everyday	0.82 (0.39–1.72)	0.71 (0.31–1.63)	0.51 * (0.28–0.91)	2.26 (0.86–5.95)	0.49 (0.03–7.47)	0.67 * (0.50–0.89)	0.95 (0.37–2.43)	0.52 ** (0.34–0.79)

OR, RR estimates and 95% CI were pooled over the 5 imputed datasets. Logistic regression, nominal logistic regression, and zero-inflated negative binomial (ZINB) models were performed to assess the risk factors of the poor oral health outcomes among people with SMI. All models contain covariates of demographics (age, sex, ethnicity, marital status, income), lifestyles (BMI, smoking status, substance misuse, physical activity, sugar intake), comorbidities (diabetes, cardiovascular disease), and oral health behaviour (dental floss use). OR, odds ratio; RR, rate ratio; DMFT, number of decayed, missing and filled teeth; DT, number of decayed teeth; MT, number of missing teeth due to decay. * *p* < 0.05, ** *p* < 0.01, *** *p* < 0.001.

## Data Availability

The NHANES 1999–2016 data is available at CDC website: https://www.cdc.gov/nchs/nhanes/index.htm, and is accessible and free to download for everyone.

## Supplementary Materials

The following supporting information can be downloaded at: https://www.mdpi.com/article/10.3390/dj12070191/s1, Figure S1: Directed acyclic graphs (DAG) for illustration of (A) severe mental illness (SMI) status on oral health outcomes, and (B) risk factors for poor oral health outcomes among people with SMI; Table S1: Full list of risk factors of poor oral health in people with severe mental illness, NHANES (*n* = 718), 1999–2016 [23,24,25,34,35,37,41,43,49,50,51,52,53,54,55,56,57,58,59,60,61].

## References

[1] Firth J., Siddiqi N., Koyanagi A., Siskind D., Rosenbaum S., Galletly C., Allan S., Caneo C., Carney R., Carvalho A.F. (2019). The Lancet Psychiatry Commission: A blueprint for protecting physical health in people with mental illness. Lancet Psychiatry.

[2] Ruggeri M., Leese M., Thornicroft G., Bisoffi G., Tansella M. (2000). Definition and prevalence of severe and persistent mental illness. Br. J. Psychiatry.

[3] Palmier-Claus J.E., Shiers D., French P., Harris R., Laverty L. (2019). Oral health in psychosis: An unmet need. Schizophr. Res..

[4] Kang J., Palmier-Claus J., Wu J., Shiers D., Larvin H., Doran T., Aggarwal V.R. (2023). Periodontal disease in people with a history of psychosis: Results from the UK biobank population-based study. Community Dent. Oral Epidemiol..

[5] Joury E., Kisely S., Watt R.G., Ahmed N., Morris A.J., Fortune F., Bhui K. (2023). Mental Disorders and Oral Diseases: Future Research Directions. J. Dent. Res..

[6] Kisely S., Quek L.H., Pais J., Lalloo R., Johnson N.W., Lawrence D. (2011). Advanced dental disease in people with severe mental illness: Systematic review and meta-analysis. Br. J. Psychiatry.

[7] Yang M., Chen P., He M.X., Lu M., Wang H.M., Soares J.C., Zhang X.Y. (2018). Poor oral health in patients with schizophrenia: A systematic review and meta-analysis. Schizophr. Res..

[8] Kilbourne A.M., Horvitz-Lennon M., Post E.P., McCarthy J.F., Cruz M., Welsh D., Blow F.C. (2007). Oral health in Veterans Affairs patients diagnosed with serious mental illness. J. Public Health Dent..

[9] Hugo F.N., Hilgert J.B., de Sousa Mda L., Cury J.A. (2009). Oral status and its association with general quality of life in older independent-living south-Brazilians. Community Dent. Oral Epidemiol..

[10] Yamamoto T., Kondo K., Aida J., Fuchida S., Hirata Y., JAGES Group (2014). Association between the longest job and oral health: Japan Gerontological Evaluation Study project cross-sectional study. BMC Oral Health.

[11] Hede B. (1995). Dental health behavior and self-reported dental health problems among hospitalized psychiatric patients in Denmark. Acta Odontol. Scand..

[12] Cook B.L., Wayne G.F., Kafali E.N., Liu Z., Shu C., Flores M. (2014). Trends in smoking among adults with mental illness and association between mental health treatment and smoking cessation. JAMA.

[13] Bahorik A.L., Newhill C.E., Queen C.C., Eack S.M. (2014). Under-reporting of drug use among individuals with schizophrenia: Prevalence and predictors. Psychol. Med..

[14] Teasdale S.B., Ward P.B., Samaras K., Firth J., Stubbs B., Tripodi E., Burrows T.L. (2019). Dietary intake of people with severe mental illness: Systematic review and meta-analysis. Br. J. Psychiatry.

[15] Scully C. (2003). Drug effects on salivary glands: Dry mouth. Oral Dis..

[16] Plemons J.M., Al-Hashimi I., Marek C.L., American Dental Association Council on Scientific Affairs (2014). Managing xerostomia and salivary gland hypofunction: Executive summary of a report from the American Dental Association Council on Scientific Affairs. J. Am. Dent. Assoc..

[17] Nielsen J., Munk-Jorgensen P., Skadhede S., Correll C.U. (2011). Determinants of poor dental care in patients with schizophrenia: A historical, prospective database study. J. Clin. Psychiatry.

[18] Turner E., Berry K., Aggarwal V.R., Quinlivan L., Villanueva T., Palmier-Claus J. (2022). Oral health self-care behaviours in serious mental illness: A systematic review and meta-analysis. Acta Psychiatr. Scand..

[19] Leite R.S., Marlow N.M., Fernandes J.K., Hermayer K. (2013). Oral health and type 2 diabetes. Am. J. Med. Sci..

[20] Vancampfort D., Correll C.U., Galling B., Probst M., De Hert M., Ward P.B., Rosenbaum S., Gaughran F., Lally J., Stubbs B. (2016). Diabetes mellitus in people with schizophrenia, bipolar disorder and major depressive disorder: A systematic review and large scale meta-analysis. World Psychiatry.

[21] Kisely S., Baghaie H., Lalloo R., Siskind D., Johnson N.W. (2015). A systematic review and meta-analysis of the association between poor oral health and severe mental illness. Psychosom. Med..

[22] Wey M.C., Loh S., Doss J.G., Abu Bakar A.K., Kisely S. (2016). The oral health of people with chronic schizophrenia: A neglected public health burden. Aust. N. Z. J. Psychiatry.

[23] National Health and Nutrition Examination Survey (2015). National Center for Health Statistics. https://www.cdc.gov/nchs/index.htm.

[24] Hong J., Whelton H., Douglas G., Kang J. (2018). Consumption frequency of added sugars and UK children’s dental caries. Community Dent. Oral Epidemiol..

[25] Rubin D.B., Schenker N. (1991). Multiple imputation in health-care databases: An overview and some applications. Stat. Med..

[26] Lamster I.B., DePaola D.P., Oppermann R.V., Papapanou P.N., Wilder R.S. (2008). The relationship of periodontal disease to diseases and disorders at distant sites: Communication to health care professionals and patients. J. Am. Dent. Assoc..

[27] Saintrain M.V., de Souza E.H. (2012). Impact of tooth loss on the quality of life. Gerodontology.

[28] Sheiham A. (2005). Oral health, general health and quality of life. Bull. World Health Organ..

[29] FDI World Dental Federation (2020). Access to oral healthcare for vulnerable and underserved populations: Adopted by the General Assembly: September 2019, San Francisco, United States of America. Int. Dent. J..

[30] Waisel D.B. (2013). Vulnerable populations in healthcare. Curr. Opin. Anaesthesiol..

[31] Elaine Muirhead V., Milner A., Freeman R., Doughty J., Macdonald M.E. (2020). What is intersectionality and why is it important in oral health research?. Community Dent. Oral Epidemiol..

[32] Kapilashrami A., Hankivsky O. (2018). Intersectionality and why it matters to global health. Lancet.

[33] Gilbody S., Peckham E., Bailey D., Arundel C., Heron P., Crosland S., Fairhurst C., Hewitt C., Li J., Members of the SCIMITAR+ Collaborative (2021). Smoking cessation in severe mental illness: Combined long-term quit rates from the UK SCIMITAR trials programme. Br. J. Psychiatry.

[34] Khan S., Khalid T., Awan K.H. (2016). Chronic periodontitis and smoking. Prevalence and dose-response relationship. Saudi Med. J..

[35] Jang A.Y., Lee J.K., Shin J.Y., Lee H.Y. (2016). Association between Smoking and Periodontal Disease in Korean Adults: The Fifth Korea National Health and Nutrition Examination Survey (2010 and 2012). Korean J. Fam. Med..

[36] (2005). Exercise benefits dental health. Br. Dent. J..

[37] Sanchez G.F.L., Smith L., Koyanagi A., Grabovac I., Yang L., Veronese N., Shin J.I., Loosemore M., Jacob L. (2020). Associations between self-reported physical activity and oral health: A cross-sectional analysis in 17,777 Spanish adults. Br. Dent. J..

[38] Virtanen J.I., Muikku T., Simila T., Cinar A.B., Pohjola V. (2019). Physical activity, BMI and oral health behaviour among adolescents: Finnish School Health Promotion Study. Eur. J. Public Health.

[39] Lin C.Y., Chen P.C., Kuo H.K., Lin L.Y., Lin J.W., Hwang J.J. (2010). Effects of obesity, physical activity, and cardiorespiratory fitness on blood pressure, inflammation, and insulin resistance in the National Health and Nutrition Survey 1999–2002. Nutr. Metab. Cardiovasc. Dis..

[40] Cepeda M.S., Weinstein R., Blacketer C., Lynch M.C. (2017). Association of flossing/inter-dental cleaning and periodontitis in adults. J. Clin. Periodontol..

[41] Sambunjak D., Nickerson J.W., Poklepovic T., Johnson T.M., Imai P., Tugwell P., Worthington H.V. (2011). Flossing for the management of periodontal diseases and dental caries in adults. Cochrane Database Syst. Rev..

[42] Rohani B. (2019). Oral manifestations in patients with diabetes mellitus. World J. Diabetes.

[43] Kinane D.F., Chestnutt I.G. (1997). Relationship of diabetes to periodontitis. Curr. Opin. Periodontol..

[44] Preshaw P.M., Alba A.L., Herrera D., Jepsen S., Konstantinidis A., Makrilakis K., Taylor R. (2012). Periodontitis and diabetes: A two-way relationship. Diabetologia.

[45] Davis K.A.S., Cullen B., Adams M., Brailean A., Breen G., Coleman J.R.I., Dregan A., Gaspar H.A., Hübel C., Lee W. (2019). Indicators of mental disorders in UK Biobank-A comparison of approaches. Int. J. Methods Psychiatr. Res..

[46] Laverty L., Harris R. (2018). Can conditional health policies be justified? A policy analysis of the new NHS dental contract reforms. Soc. Sci. Med..

[47] Adams C.E., Wells N.C., Clifton A., Jones H., Simpson J., Tosh G., Callaghan P., Liddle P., Guo B., Furtado V. (2018). Monitoring oral health of people in Early Intervention for Psychosis (EIP) teams: The extended Three Shires randomised trial. Int. J. Nurs. Stud..

[48] Kuo M.W., Yeh S.H., Chang H.M., Teng P.R. (2020). Effectiveness of oral health promotion program for persons with severe mental illness: A cluster randomized controlled study. BMC Oral Health.

[49] Grossi S.G., Zambon J.J., Ho A.W., Koch G., Dunford R.G., Machtei E.E., Norderyd O.M., Genco R.J. (1994). Assessment of risk for periodontal disease. I. Risk indicators for attachment loss. J. Periodontol..

[50] Persson G.R., Persson R.E., Hollender L.G., Kiyak H.A. (2004). The impact of ethnicity, gender, and marital status on periodontal and systemic health of older subjects in the Trials to Enhance Elders’ Teeth and Oral Health (TEETH). J. Periodontol..

[51] Weatherspoon D.J., Borrell L.N., Johnson C.W., Mujahid M.S., Neighbors H.W., Adar S.D. (2016). Racial and Ethnic Differences in Self-Reported Periodontal Disease in the Multi-Ethnic Study of Atherosclerosis (MESA). Oral Health Prev. Dent..

[52] Paulander J., Axelsson P., Lindhe J. (2003). Association between level of education and oral health status in 35-, 50-, 65- and 75-year-olds. J. Clin. Periodontol..

[53] National Institute of Dental Research (U.S.), Epidemiology and Oral Disease Prevention Program (1987). Oral Health of United States Adults: The National Survey of Oral Health in U.S. Employed Adults and Seniors, 1985–1986: National Findings.

[54] Martinez-Herrera M., Silvestre-Rangil J., Silvestre F.J. (2017). Association between obesity and periodontal disease. A systematic review of epidemiological studies and controlled clinical trials. Med. Oral Patol. Oral Cir. Bucal..

[55] Albandar J.M., Streckfus C.F., Adesanya M.R., Winn D.M. (2000). Cigar, pipe, and cigarette smoking as risk factors for periodontal disease and tooth loss. J. Periodontol..

[56] Shepherd S. (2011). Alcohol consumption a risk factor for periodontal disease. Evid. Based Dent..

[57] Arora G.F.R. (2021). Oral Health and Addiction: Consequences of Substance Use. Textbook of Addiction Treatment.

[58] Moynihan P.J. (1995). The relationship between diet, nutrition and dental health: An overview and update for the 90s. Nutr. Res. Rev..

[59] Sanz M., Del Castillo A.M., Jepsen S., Gonzalez-Juanatey J.R., D’Aiuto F., Bouchard P., Chapple I., Dietrich T., Gotsman I., Graziani F. (2020). Periodontitis and Cardiovascular Diseases. Consensus Report. J. Clin. Periodontol..

[60] Thomson W.M., Williams S.M., Broadbent J.M., Poulton R., Locker D. (2010). Long-term dental visiting patterns and adult oral health. J. Dent. Res..

[61] Kumar S., Tadakamadla J., Johnson N.W. (2016). Effect of Toothbrushing Frequency on Incidence and Increment of Dental Caries: A Systematic Review and Meta-Analysis. J. Dent. Res..

