# Supplementary material for: Investigating the Relationship between Oral Health and Severe Mental Illness: Analysis of NHANES 1999–2016
Source: Dent J (Basel). 2024 Jun 24;12(7):191. doi: 10.3390/dj12070191 (PMC11276391; doi:10.3390/dj12070191)
Supplement: Supplementary file 1 [file dentistry-12-00191-s001.zip › dentistry-3007962-supplementary.pdf]

Supplementary Materials for "Oral health and severe mental illness: Analysis of NHANES 1999-2016"

Supplement Methods

Study design: The study followed STROBE guidelines. Study participants came from the cross sectional NHANES 1999-2016, a national survey designed to assess the health and nutritional status for the non-institutionalised United States population using a stratified, multistage, probability sampling design. NHANES has used the same survey structure and collected data in two-year cycles since 1999/2000. It consists of extensive anthropometric, socioeconomic, health and dental related examinations and questionnaires, as well as laboratory testing for biomarkers. Height, weight and waist circumference were measured onsite by trained examiners; dental-related measures were taken by trained dental survey staff and periodically quality controlled by a second "gold standard" examiner. The methods for NHANES are available elsewhere [23].

Study participants: We extracted data from nine NHANES surveys (1999-2016). The resulting sample from NHANES for participants over 18 years old was 53,348 participants (25,709 men and 27,639 women). The sample sizes for each cohort was: 1999-2000 *n*=9,965, 2001-02 *n*=11,039, 2003-04 *n*=10,122, 2005-06 *n*=10,348, 2007-08 *n*=10,149, 2009-10 *n*=10,537, 2011-12 *n*=9,756, 2013-14 *n*=10,175, 2015-16 *n*=9,971. NHANES does not contain clinical diagnoses of mental illness, so participants' prescription medicine in the past month were extracted and people with SMI were identified based on the type of medication that they were taking. If participants reported to take one or more medications for treating SMI, we considered them to have SMI. Validation of drug code is via ICD-10-CM code (F20, F29, F31.9), and mental illnesses such as bipolar and psychosis were identified. The generic drug code and name extracted from NHANES data is 'RXDDRGID', and the drug related to SMI are: c00251 Antipsychotics-unspecified, d04825 Aripiprazole, d00064 Chlorpromazine, d00199 Clozapine, d00237 Fluphenazine, d00027 Haloperidol, d00061 Lithium, d00897 Loxapine, d07705 Lurasidone, d04050 Olanzapine, d06297 Paliperidone, d00855 Perphenazine, d00898 Pimozide, d04220 Quetiapine, d03180 Risperidone, d00389 Thioridazine, d00890 Trifluoperazine, and d04747 Ziprasidone.

Oral health outcome measures: Oral health outcomes included dentition, dental caries, periodontal status, and self-reported oral health status.

- Dentition was measured by the number of teeth (0, edentulous; 32, full dentition) by trained and calibrated health technologists. Tooth loss due to traumatic injuries were excluded in the analyses because we focused on oral disease. For the analysis, tooth loss status was derived from the number of teeth and categorized as 'no loss, 1-10, 11-20, 21-31 and edentulous'. Dentition data was available in NHANES 1999-2004, 2011-2016.
- Dental caries is reported as the number of DMFT and was derived from the coronal caries status of each tooth coded as "missing due to dental disease, permanent tooth with a restored surface condition, permanent root tip is present but no restorative replacement is present, missing due to dental disease but replaced by a removable restoration, missing due to dental disease but replaced by a fixed restoration, permanent tooth with a dental carious surface condition". The number of coronal decayed teeth (DT) is derived from the same tooth coronal caries status when coded as "permanent tooth with a dental carious surface condition", and number of missing teeth (MT) due to decay is obtained when the coronal caries status was coded as "missing due to dental disease, missing due to dental disease but replaced by a removable restoration, missing due to dental disease but replaced by a fixed restoration". Dental caries data was available in NHANES 1999-2004, 2011-2016.
- Periodontal status was classified as 'none, mild, moderate and severe' by using a standard definition for surveillance of periodontitis[1]. Severe periodontitis was defined as two or more sites with >=6mm clinical attachment loss and one or more sites with >=5mm pocket depth (not on the same tooth). Moderate periodontitis was defined as two or more sites with >=4mm clinical attachment loss or two or more sites with pocket depth of >=5mm

(not on the same tooth). Mild periodontitis was defined as two or more sites with >=3mm clinical attachment loss and two or more sites with >=4mm pocket depth, or one site with >=5mm pocket depth (not on the same tooth). Periodontal measurements were available in 1999-2004 and 2009-2014. In NHANES 2009-2014, periodontal measurements were made at six sites per tooth (mesio-, mid-,and disto-buccal; mesio-, mid-, and-distol-lingual) for all teeth except third molars, while in NHANES 1999-2004 periodontal measurements were made at only two sites per tooth (mid-buccal, mesio-buccal). In order to make the classification of the periodontal status consistent across cohorts, the two sites (mid-buccal, mesio-buccal) were used in the analysis.

- Self-reported oral health included self-rated oral health status and mouth ache. Self-rated oral health status was obtained in the interview question “How would you describe the condition of your teeth and gums?”, with the options ‘Excellent’, ‘very good’, ‘good’, ‘fair’, and ‘poor’. Mouth ache was obtained from the interview question “How often during the last year has you had painful aching anywhere in your mouth?” with options ’very often’, ‘fairly often’, ‘occasionally’, ‘hardly ever’, and ‘never’. Self-reported oral health status was available in 1999-2002 and 2005-2016; mouth ache was available in 2005-2008 and 2011-2016.

Exposures: The following set of variables were included in the analyses which were coded based on original NHANES variable or further categorized by evidence based literature or guideline:

- Demographic variables: Age (18 and above, scale) [49], sex (male or female) [50], ethnicity (white or other ethnicity) [50,51], education qualification (high school or below, college or above) [52], marital status (not married, married) [52], and ratio of family income to poverty (scale) [53].
- Anthropometric measures: body mass index (BMI, kg/m^2), waist group (low (men <=94cm , women<=80cm), high (men 94-102cm, women 80-88cm), very high (men >102cm, women >88cm) [54].
- Lifestyle factors: smoking status (non-smoker, ex-smoker, current smoker) [34], cigarette number in the past 30 days (scale) [35,55], had at least 12 alcohol drinks in one year (yes or no) [35], substance misuse (ever used cocaine or other street drug, yes or no) [56], moderate physical activity over past 30 days (yes or no) [57]; sugar intake (gram), carbohydrate intake (gram), and energy intake (KCAL) [58].
- Comorbidities (yes or no): cardiovascular disease (including with at least one of congestive heart failure, coronary heart disease, angina, heart attack, and stroke) [43] and diabetes [59].
- Dental hygiene behaviour: Time since last dental visit (less than 1 year, over 1 year, never) [60], tooth brush frequency per day (once or less, twice or more) [61], use dental floss (no, not everyday, everyday) [41]. Data for time since last dental visit was available in NHANES 1999-2004 and 2013-2016; tooth brushing frequency data was available in 2013-2016; and dental floss data was available in 2009-2016.

Statistical analyses: Descriptive statistics compared people with and without SMI, concerning demographics, anthropometrics, lifestyles, comorbidities, dental hygiene behaviour, and all oral health outcomes (dentition, dental caries, periodontal status, and self-reported oral health status). Continuous variables were presented as mean (*SD*) or median (interquartile range), and categorical variables were reported as frequency (%). The general population were matched to people with SMI on a ratio 1 to 3 based on their age and gender because the distribution for people with and without SMI were different in the original dataset (people with SMI were of older age than people without SMI), and matching the sample provided comparable results.

For statistical modelling, self-rated oral health was further grouped as ‘excellent or very good or good’ and ‘fair or poor’, ache in mouth as ‘never or hardly ever’ and ‘occasionally to very often’, and periodontal status was further grouped as ‘none’, ’mild to moderate’, and ’severe’ to ensure sufficient events in each category. Smoking was grouped as two categories (non-smoker, ever smoker) as was dental visiting (less than 1 year, more than 1 year or never). Alcohol and energy intake were excluded in the statistical model because they were not significant between people with and without SMI. Education was

highly correlated with family income and therefore only the latter was included in the models. Cigarette number only applied to smokers so it was excluded in the modelling process. Tooth brushing frequency had only a very small number of responses (<1% of the total sample) in both groups and was excluded. The ordinal variables of dentition (tooth loss) and periodontal status necessitated ordinal regression models. Self-reported oral health (self-rated oral health status, mouth ache) were binary so logistic regression models were applied. First, a univariable model with one of the ordinal oral health outcomes as dependent variable and group (SMI or non-SMI) as independent variable was performed. Then multivariable models were performed with gradual adjustment of demographic, lifestyles, comorbidities, and dental hygiene behaviours. Similarly, zero-inflated negative binomial (ZINB; see supplementary material 1) models were used to compare SMI and non-SMI population on dental caries (DMFT, DT and MT), because dental caries variables have excess zeros and follows a negative binomial distribution. A ZINB model is a 2-part model, with the logit model predicting excessive zeros, and negative binomial model predicting the counts [24]. Similar approaches for the univariable ZINB model and multivariable ZINB model were applied for DMFT, DT, and MT respectively.

When investigating predictors of oral health status in people with SMI alone, ordinal regression, logistic regression, and ZINB models were used for ordinal, binary, and scale oral outcomes respectively with similar approaches. Missing data were imputed five times through multiple imputation by chained equations according to the distribution of the imputed variables. Pooled modelling estimates and accompanying standard errors (SE) were generated according to Rubin's rules [25]. Data was also analyzed on complete case and sensitivity analyses were conducted. Statistical analyses were performed in R version 3.4.1 (https://cran.r-project.org/) with various packages (e.g. MASS, MICE, Amelia).

Table S1. Full list of risk factors of poor oral health in people with severe mental illness, NHANES (n = 718), 1999-2016.

	**Ordinal Oral health outcomes, OR (95% CI)**				**Numeric oral health outcomes, OR and RR (95% CI)**					
	Self-rated oral health	Ache in mouth	Tooth loss number (grouped)	Periodontal disease severity	DMFT		DT		MT	
					OR (95% CI)	RR (95% CI)	OR (95% CI)	RR (95% CI)	OR (95% CI)	RR (95% CI)
n	637	422	718	285	718		718		718	
**Demographics**										
Age	1.01 (0.99-1.03)	1.00 (0.98-1.03)	1.07 *** (1.05-1.09)	1.03 (1.00-1.06)	0.98 (0.97-1.00)	1.03*** (1.02-1.03)	1.01 (0.99-1.03)	1.01 (1.00 - 1.02)	0.96*** (0.94-0.98)	1.04*** (1.03-1.06)
Sex, male	1.14 (0.76-1.72)	0.74 (0.44-1.24)	0.76 (0.53-1.09)	1.07 (0.59-1.94)	1.01 (0.68-1.49)	0.96 (0.81-1.13)	0.98 (0.66-1.47)	1.05 (0.83 - 1.33)	0.95 (0.60-1.51)	0.85 (0.62-1.16)
Ethnicity, white	1.10 (0.72-1.67)	1.35 (0.79-2.29)	1.40 (0.98-2.00)	0.62 (0.35-1.10)	1.30 (0.87-1.93)	1.25** (1.06-1.48)	1.24 (0.83-1.87)	1.18 (0.94 - 1.48)	1.76** (1.09-2.77)	1.57** (1.15-2.16)
Marital status, not married	1.07 (0.66-1.74)	1.02 (0.57-1.84)	0.98 (0.63-1.51)	1.04 (0.53-2.05)	1.08 (0.68-1.73)	1.03 (0.84-1.26)	1.28 (0.79-2.07)	1.00 (0.75 - 1.33)	0.98 (0.57-1.71)	1.12 (0.76-1.64)
Ratio of family income to poverty	0.84 * (0.72-0.99)	0.74 ** (0.59-0.91)	0.77 *** (0.67-0.89)	0.90 (0.71-1.13)	1.14 (0.98-1.33)	0.94 (0.87-1.00)	1.01 (0.87-1.18)	1.05 (0.95 - 1.16)	1.31* (1.08-1.58)	0.82 (0.72-0.93)
**Lifestyles**										
BMI	1.01 (0.98-1.04)	1.03 (0.99-1.06)	1.00 (0.97-1.02)	0.97 (0.93-1.01)	1.02 (0.99-1.05)	0.99 (0.98-1.01)	1.01 (0.98-1.04)	1.00 (0.98 - 1.02)	1.02 (0.99-1.05)	1.00 (0.98-1.02)

Smoking status, ever smoker	1.36 (0.85-2.18)	0.70 (0.39-1.25)	2.62 *** (1.72-3.99)	1.23 (0.63-2.38)	0.95 (0.60-1.49)	1.23* (1.02-1.49)	1.27 (0.80-2.00)	0.92 (0.72 - 1.17)	0.72 (0.42-1.24)	1.79** (1.24-2.58)
Substance misuse (cocaine/heroin), yes	1.24 (0.80-1.92)	1.21 (0.70-2.10)	0.95 (0.65-1.39)	0.89 (0.48-1.66)	1.21 (0.79-1.84)	0.96 (0.81-1.14)	1.01 (0.65-1.55)	0.93 (0.72 - 1.19)	1.17 (0.72-1.91)	0.87 (0.64-1.19)
Physical activity, yes	0.54 ** (0.34-0.86)	1.28 (0.73-2.25)	0.74 (0.48-1.12)	1.22 (0.62-2.40)	0.87 (0.56-1.34)	1.01 (0.84-1.22)	1.00 (0.64-1.56)	1.07 (0.83 - 1.38)	0.85 (0.51-1.43)	0.93 (0.66-1.33)
Sugar intake, every increase of 100g	1.17 (0.91-1.50)	1.23 (0.86-1.75)	1.04 (0.82-1.31)	0.96 (0.66-1.40)	1.07 (0.82-1.39)	1.03 (0.93-1.14)	1.06 (0.83-1.33)	1.08 (0.93 - 1.25)	1.18 (0.87-1.60)	1.03 (0.85-1.25)
**Comorbidities**										
Diabetes, yes	1.58 (0.85-2.92)	1.16 (0.58-2.33)	2.05 * (1.18-3.55)	1.36 (0.61-3.03)	0.83 (0.46-1.50)	1.24 (0.99-1.56)	1.32 (0.71-2.46)	1.08 (0.74 - 1.59)	0.79 (0.41-1.51)	1.52* (1.03-2.24)
Cardiovascular disease, yes	1.68 (0.86-3.29)	1.48 (0.72-3.05)	1.20 (0.68-2.11)	1.10 (0.43-2.85)	1.50 (0.81-2.79)	1.03 (0.79-1.34)	1.40 (0.72-2.71)	0.92 (0.61 - 1.38)	1.35 (0.67-2.71)	0.91 (0.59-1.43)
**Dental hygiene behaviour**										
Last dental visit, over 1 year or never	1.12 (0.73-1.73)	0.94 (0.46-1.90)	0.90 (0.59-1.38)	1.10 (0.52-2.31)	1.08 (0.60-1.92)	0.92 (0.76-1.10)	1.08 (0.60-1.93)	0.83 (0.63 - 1.10)	1.33 (0.70-2.54)	1.10 (0.80-1.51)
Use dental floss in the past 7 days										
No (ref)	1	1	1	1	1	1	1	1	1	1

Not everyday	1.22 (0.70-2.14)	1.00 (0.49-2.02)	0.55 (0.26-1.17)	1.00 (0.42-2.36)	1.32 (0.78-2.25)	0.86 (0.66-1.13)	0.92 (0.53-1.59)	1.13 (0.83 - 1.53)	1.37 (0.68-2.74)	0.61 (0.36-1.03)
Everyday	0.79 (0.38-1.67)	0.76 (0.33-1.72)	0.50 * (0.28-0.90)	1.66 (0.55-4.98)	0.86 (0.40-1.89)	0.71* (0.53-0.96)	0.86 (0.39-1.91)	1.19 (0.83 - 1.69)	0.95 (0.37-2.43)	0.52** (0.34-0.79)

[a] OR, RR estimates and 95% CI were pooled over the 5 imputed datasets.

[b] Logistic regression, nominal logistic regression, and zero-inflated negative binomial (ZINB) models were performed to assess the risk factors of the poor oral health outcomes among people with SMI. All models contains covariates of demographics (age, sex, ethnicity, marital status, income), lifestyles (BMI, smoking status, substance misuse, physical activity, sugar intake), comorbidities (diabetes, cardiovascular disease), and oral health behaviour (dental floss use).

OR, odds ratio; RR, rate ratio; DMFT, number of decayed, missing and filled teeth; DT, number of decayed teeth; MT, number of missing teeth due to decay.

Figure S1. Directed acyclic graphs (DAG) for illustration of (A) severe mental illness (SMI) status on oral health outcomes, and (B) risk factors for poor oral health outcomes among people with SMI.

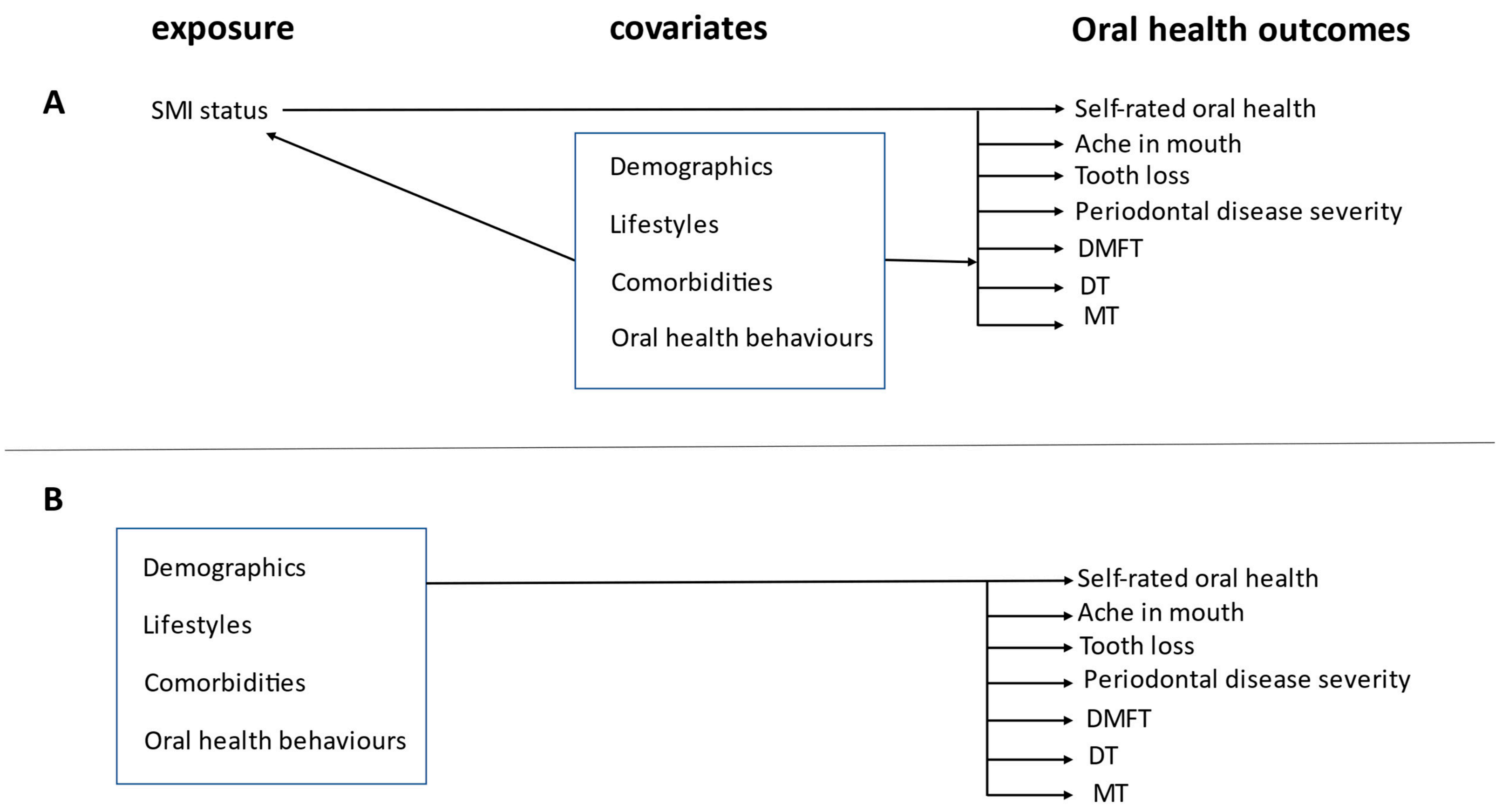

Note: Demographics: age, sex, ethnicity, marital status, income; lifestyles: BMI, smoking status, substance misuse, physical activity, sugar intake; comorbidities: diabetes, cardiovascular disease; and oral health behaviour: dental visit (A) and dental floss use (A,B). DMFT, decayed, missing and filled teeth. DT, decayed teeth. MT, missing teeth.

References:

49 Grossi SG, Zambon JJ, Ho AW, et al. Assessment of risk for periodontal disease. I. Risk indicators for attachment loss. *J Periodontol.* 1994;65(3):260-267.

50 Persson GR, Persson RE, Hollender LG, Kiyak HA. The impact of ethnicity, gender, and marital status on periodontal and systemic health of older subjects in the Trials to Enhance Elders' Teeth and Oral Health (TEETH). *J Periodontol.* 2004;75(6):817-823.

51 Weatherspoon DJ, Borrell LN, Johnson CW, Mujahid MS, Neighbors HW, Adar SD. Racial and Ethnic Differences in Self-Reported Periodontal Disease in the Multi-Ethnic Study of Atherosclerosis (MESA). *Oral Health Prev Dent.* 2016;14(3):249-257.

52 Paulander J, Axelsson P, Lindhe J. Association between level of education and oral health status in 35-, 50-, 65- and 75-year-olds. *J Clin Periodontol.* 2003;30(8):697-704.

53 Miller AJ BJ, Carlos JP, Brown LJ, Löe H. Oral Health of United States Adults: National Findings. *Bethesda, MD: National Institute of Dental Research, NIH.* 1987.

54 Martinez-Herrera M, Silvestre-Rangil J, Silvestre FJ. Association between obesity and periodontal disease. A systematic review of epidemiological studies and controlled clinical trials. *Med Oral Patol Oral Cir Bucal.* 2017;22(6):e708-e715.

55 Albandar JM, Streckfus CF, Adesanya MR, Winn DM. Cigar, pipe, and cigarette smoking as risk factors for periodontal disease and tooth loss. *J Periodontol.* 2000;71(12):1874-1881.

56 Shepherd S. Alcohol consumption a risk factor for periodontal disease. *Evid Based Dent.* 2011;12(3):76.

57 Arora G. FR. Oral Health and Addiction: Consequences of Substance Use. *Textbook of Addiction Treatment.* 2021.

58 Moynihan PJ. The relationship between diet, nutrition and dental health: an overview and update for the 90s. *Nutr Res Rev.* 1995;8(1):193-224.

59 Sanz M, Del Castillo AM, Jepsen S, et al. Periodontitis and Cardiovascular Diseases. Consensus Report. *Glob Heart.* 2020;15(1):1.

60 Thomson WM, Williams SM, Broadbent JM, Poulton R, Locker D. Long-term dental visiting patterns and adult oral health. *J Dent Res.* 2010;89(3):307-311.

61 Kumar S, Tadakamadla J, Johnson NW. Effect of Toothbrushing Frequency on Incidence and Increment of Dental Caries: A Systematic Review and Meta-Analysis. *J Dent Res.* 2016;95(11):1230-1236.
